# Supplementary material for: Efficacy of anti-vascular endothelial growth factor agents for treating neovascular age-related macular degeneration in vitrectomized eyes
Source: PLoS One. 2021 Jun 10;16(6):e0252006. doi: 10.1371/journal.pone.0252006 (PMC8191940; doi:10.1371/journal.pone.0252006)
Supplement: Attachment — Submitted filename: PLOS ONE_PONE_D_20_07419.docx [file pone.0252006.s001.docx]

Number: PONE-D-20-07419:

A small amount of patients and eyes but still clinically a very interesting retrospective original work.

A control group of non-vitrectomized eyes would strengthen this work.

For review comments see below.

**Abstract:**

Line 18: intravitreal anti-vascular endothelial growth factor is shortened as (VEGF). Shouldn`t it be (anti-VEGF)?

Line 27: Do you have proof for this no A-V communication in group A. In table 1 31 eyes in group A are pseudophakic. Are you shure that there has not been any capsule breaks or rupture in these cases?

Line 31, 35: “multiple injections” – how many injections for how many months or years?

Line 38-40: Please reformulate the conclusion.

**Introduction:**

Line 48, please add “used”: …are anti-VEGF agents “used” for the treatment of nAMD.

Line line 50: … elderly patients “often” receive vitrectomy… Do you have proof for this statement? How often is vitrectomy actually done?

Line 57: How about clearance? Could you comment on this?

Line 59-63: There have been clinical studies of macular edema due to diabetic retinopathy with a larger amount of eyes, 25 eyes in 2015 and 33 eyes in 2017. Could you comment on this? How can you be sure that all your patients have underwent total vitrectomy and not core vitrectomy? Is this essential for your statement of A-V communication? Is this essential for your conclusion?

**Materials and methods:**

Line 69-70: In which years did you collect data? Where all patients treatment naïve nAMD before vitrectomy?

Line 76: How many injections for how many months or years? Had any of your patients received laser before anti-VEGF treatment?

Line 79: (3) Please explain this sentence. Is the purpose of anti-VEGF not to dry the macula?

Line 83-84: Do you have proof for this A-V communication?

Line 85: “Before treatment..” – which treatment vitrectomy or anti-VEGF treatment?

Line 90: Which VA did you use, the best measured VA or an average or?

Line 95: When was the first anti-VEGF injection given? Did you always use monthly injections, or PRN injections or?

Line 108: “… three groups…” Weren`t there only 2 groups, group A and B?

**Results:**

Table 1: Please comment in your discussion on the amount of patients having diabetes mellitus and cancer. Could this influence your results? Did you switch between different types of anti-VEGF?

Figure 1: N=141? On line 69 under “Materials and Methods” you have reviewed 207 medical records? In the same figure you state that “Group A: With A-V communication” and Goup B: Without A-V communication? Isn`t it the opposite? Why did you exclude N=11 eyes receiving anti-VEGF for maintenance?

**Discussion:**

It could improve the work having a control group. Could you comment on that?

Line 245: What would be the normal improvement of vision in non-vitrectomized eyes having nAMD? Did your vitrectomized patients receive more injection than in general?
