# Supplementary material for: Efficacy of anti-vascular endothelial growth factor agents for treating neovascular age-related macular degeneration in vitrectomized eyes
Source: PLoS One. 2021 Jun 10;16(6):e0252006. doi: 10.1371/journal.pone.0252006 (PMC8191940; doi:10.1371/journal.pone.0252006)
Supplement: Attachment — Submitted filename: Response_to_Reviewers_20200907_PlosOne.docx [file pone.0252006.s002.docx]

September 8, 2020

Simone Donati, M.D.

Academic Editor

*PLOS ONE*

Dear Dr. Donati,

Thank you for inviting us to submit a revised draft of our manuscript entitled, "Efficacy of anti-vascular endothelial growth factor agents for treating neovascular age-related macular degeneration in vitrectomized eyes" to *PLOS ONE*. We also appreciate the time and effort you and the reviewer dedicated to providing insightful feedback about strengthening. Thus, it is with great pleasure that we resubmit our article for further consideration. We have incorporated changes that reflect the detailed suggestions you have graciously provided. We hope that our edits and the responses we provide below satisfactorily address all the issues and concerns you and the reviewer noted.

To facilitate review of our revisions, here we provide point-by-point response to the questions and comments delivered in your letter dated Aug 05, 2020.

**Reviewer #1: Number: PONE-D-20-07419:**

A small amount of patients and eyes but still clinically a very interesting retrospective original work.

A control group of non-vitrectomized eyes would strengthen this work.

For review comments see below.

🡪 Thank you for providing these insights. We also thought it would be better to select a comparative group. However, it was difficult to design a comparative (non-vitrectomized) group with equivalent characteristics since the patients in our study had retinal pathologies in addition to nAMD. We added this point as a limitation (p. 14, lines 279–281). We also supplemented the lack of a comparative group by adding the results of real-world studies (p.13, lines 254–256).

***(p. 14, lines 279–281)*** It was difficult to design a comparative group since several patients in our study had retinal pathologies such as an epiretinal membrane or macular hole in addition to nAMD.

***(p.13, lines 254–256)*** In a recent real-world study of anti-VEGF with ranibizumab treatment, the mean VA change was -0.062 ± 0.33 logMAR at 1 year from baseline in treatment-naïve patients with a mean of 5.0 ± 2.7 injections.

*Abstract:*

Line 18: intravitreal anti-vascular endothelial growth factor is shortened as (VEGF). Shouldn`t it be (anti-VEGF)?

🡪 Since we abbreviated only “vascular endothelial growth factor” and not “anti”, we wrote only VEGF in parentheses. But it can be confusing as your you have mentioned in your comment; therefore, we have replaced the term “VEGF” with "anti-VEGF" (p. 2, lines 18–20).

***(p. 2, lines 18–20)*** Evaluate the efficacy of intravitreal anti-vascular endothelial growth factor (anti-VEGF) agents for treatment of neovascular age-related macular degeneration (nAMD) in vitrectomized eyes.

Line 27: Do you have proof for this no A-V communication in group A. In table 1 31 eyes in group A are pseudophakic. Are you sure that there has not been any capsule breaks or rupture in these cases?

🡪 Several patients in group A underwent Nd:YAG laser posterior capsulotomy. However, in this case, there was a little gap between the remaining posterior capsule and the intraocular lens (IOL) that could still could act as aqueous-vitreous barrier. Despite this, drug diffusion and a lack of A-V communication could not be completely prevented. Therefore, instead of using the term “with or without A-V communication,” we directly described the presence or absence of the posterior capsule (i.e., with or without posterior capsule). We also now show the overall result without separating the patients into two groups and then show the subgroup analysis results according to presence or absence of the posterior capsule. We incorporated this throughout our paper.

***(p. 2, lines 26–29)*** A total of 44 eyes from 44 patients were included. The mean central foveal thickness (CFT) was 478.50 ± 156.93 μm at baseline, 414.25 ± 143.55 μm (86.6% of baseline) at 1 month after first injection (P < 0.001), and 386.75 ± 141.45 μm (80.8% of baseline) after monthly multiple injections (2.30 ± 1.07; range, 1–5) (P < 0.001).

***(p. 2, lines 32–36)*** In the subgroup analysis, the CFT of eyes with and without posterior capsule decreased at 1 month after both the first injection (85.8% and 91.6% of baseline, respectively) and monthly multiple injections (79.8% and 87.4% of baseline, respectively). The CFT decreased significantly more in eyes with posterior capsule (P < 0.001).

Line 31, 35: “multiple injections” – how many injections for how many months or years?

🡪 Thank you for raising this important query. Patients with nAMD underwent a mean 2.30 intravitreal anti-VEGF injections (range, 1–5). The mean, standard deviation, and range are described in parentheses (p. 2, lines 26–29). Since monthly means every month, the injection period was 1–5 months. This is also described as “Number of monthly multiple injections” in Table 1.

***(p. 2, lines 26–29)*** The mean central foveal thickness (CFT) was 478.50 ± 156.93 μm at baseline, 414.25 ± 143.55 μm (86.6% of baseline) at 1 month after the first injection (P < 0.001), and 386.75 ± 141.45 μm (80.8% of baseline) after monthly multiple injections (2.30 ± 1.07; range, 1–5) (P < 0.001).

Line 38-40: Please reformulate the conclusion.

🡪 We agree with you and have revised the conclusion accordingly (p. 2, lines 38–39).

***(p. 2, lines 38–39)*** Monthly injections of intravitreal anti-VEGF agents induced favorable anatomical improvement and vision maintenance in vitrectomized eyes with nAMD.

*Introduction:*

Line 48, please add “used”: …are anti-VEGF agents “used” for the treatment of nAMD.

🡪 We have incorporated your comments by adding “used” accordingly (p. 3, line 47).

**(p. 3, line 47)** … anti-VEGF agents used for the treatment of nAMD, for which …

Line line 50: … elderly patients “often” receive vitrectomy… Do you have proof for this statement? How often is vitrectomy actually done?

🡪 The retinal diseases that require vitrectomy, such as retinal detachment, vitreous hemorrhage, complicated cataract surgery, macular hole, or macular epiretinal membrane, are usually more prevalent in elderly than in young people. We have added new references and revised the text accordingly (p. 3, lines 49–51).

***(p. 3, lines 49–51)*** In real-world clinical practice, the retinal diseases that require vitrectomy, such as retinal detachment, vitreous hemorrhage, complicated cataract surgery, macular hole, or macular epiretinal membrane, are usually more prevalent in elderly than in young people.[7-12]

Line 57: How about clearance? Could you comment on this?

🡪 Intraocular half-lives of anti-VEGF depended on elimination (clearance), rather than degradation. We have reflected on your comment by revising this sentence (p. 3, line 56). Clearance was also covered in the discussion (p. 12, lines 211–225).

***(p. 3, line 56)*** …retinal specialists argue that the intraocular clearance of intravitreal anti-VEGF would increase.…

***(p. 12, lines 211–225)*** It is now controversial whether the clearance of intravitreal anti-VEGF agents is increased in vitrectomized eyes. Two studies of bevacizumab and ranibizumab treatment after vitrectomy in rabbit eyes reported that the half-lives of these drugs did not differ from those without vitrectomy.[13,14] However, these studies were limited since complete PVD was impossible and the remnant vitreous shell could have affected the pharmacokinetics of the anti-VEGF agents. In contrast, another two studies of bevacizumab, ranibizumab, and aflibercept treatment after vitrectomy with lensectomy in macaque eyes reported that the half-lives of these drugs were decreased compared to those in non-vitrectomized eyes.[16,28] The combined lensectomy could significantly affect intravitreal clearance because it combines the vitreous cavity and anterior chamber into a single compartment; in particular, drug clearance through the anterior chamber could be altered after combined lensectomy and vitrectomy.[13,14] Christoforidis et al showed that the half-lives of ranibizumab and bevacizumab decreased after vitrectomy without a lensectomy in rabbit eyes using a radiolabeled agent, but this study was limited since it did not determine the effect of uncoupling between iodine-124 and anti-VEGF agents.[13-15]

Line 59-63: There have been clinical studies of macular edema due to diabetic retinopathy with a larger amount of eyes, 25 eyes in 2015 and 33 eyes in 2017. Could you comment on this?

🡪 Thank you for your suggestion. Some of these papers include:

*1) Intravitreal ranibizumab for diabetic macular oedema in previously vitrectomized eyes (Laugesen CS, 2017)*

*2) Diabetic Retinopathy Clinical Research Network. Ranibizumab plus prompt or deferred laser for diabetic macular edema in eyes with vitrectomy before anti-vascular endothelial growth factory therapy (Bressler SB, 2015)*

We have reflected on your comment by adding these two papers to the reference list and revising the sentence accordingly (p. 3, lines 59–63).

***(p. 3, lines 59–63)*** Several clinical studies reported on the effect of intravitreal anti-VEGF in vitrectomized eyes, but these were only small case series (nAMD, 4 eyes), cohorts of patients with pathologies other than nAMD (macular edema due to diabetic retinopathy or retinal vein occlusion), or cases of patients who underwent core, rather than total vitrectomy.[18-23]

How can you be sure that all your patients have underwent total vitrectomy and not core vitrectomy?

🡪 We always induce posterior vitreous detachment (PVD) during vitrectomy and remove as much vitreous as possible so the vitreous cortex acts as a barrier to drug clearance. We have incorporated your comment by adding this point accordingly (p. 4, line 92).

***(p. 4, line 92)*** The vitreous cortex was removed as much as possible.

Is this essential for your statement of A-V communication?

🡪 Total vitrectomy is not essential for A-V communication because it is related to the presence of the aqueous-vitreous barrier (i.e., posterior capsule) regardless of vitrectomy. Instead of using the term “A-V communication,” we have directly described the presence or absence of the posterior capsule (with or without the posterior capsule) as mentioned above (question for line 27).

Is this essential for your conclusion?

🡪 Total vitrectomy is essential to our conclusion since the remnant vitreous cortex interferes with evaluation of the vitreous clearance. We have clarified that point (p. 12, lines 215–216; p. 15, lines 284–286).

***(p. 12, lines 215–216)*** However, these studies were limited as complete PVD was impossible and the remnant vitreous shell could have affected the pharmacokinetics of the anti-VEGF agents.

***(p. 14-15, lines 284–286)*** Despite these limitations, this is the first retrospective cohort study to show the effect of intravitreal anti-VEGF agents after complete PVD induction and sufficient peripheral vitrectomy in human eyes.

*Materials and methods:*

Line 69-70: In which years did you collect data?

🡪 Thank you for noting this. We collected data from March 2009 to December 2018. We have incorporated your comment by adding the data collection period accordingly (p. 4, lines 69–70).

***(p. 4, lines 69–70)*** We retrospectively reviewed the electronic medical records of 141 vitrectomized eyes of 141 patients with nAMD treated between March 2009 and December 2018.

Where all patients treatment naïve nAMD before vitrectomy?

🡪 As shown in Table 1, 17 patients were treatment-naïve (p. 7, Table 1).

Line 76: How many injections for how many months or years?

🡪 We did not limit the number of intravitreal injections or duration of treatment. Patients received intravitreal anti-VEGF injections repeatedly until the macular fluid disappeared regardless of the number of injections. We have revised the sentence for clarity accordingly (p. 5, lines 98–101).

***(p. 5, lines 98–101)*** Based on OCT findings, patients who showed an insufficient response were given monthly multiple injections until a sufficient decrease in intraretinal or subretinal fluid – i.e. fluid-free macula – was achieved.

Had any of your patients received laser before anti-VEGF treatment?

🡪 Two patients received photodynamic therapy (PDT) once each before enrolling in our study. No patients received PDT during the study period. We reflected your comment by adding this point to the Results section (p. 6, lines 130–131).

***(p. 6, lines 130–131)*** 2 eyes (4.5%) received photodynamic therapy before enrolling in our study.

Line 79: (3) Please explain this sentence. Is the purpose of anti-VEGF not to dry the macula?

🡪 Thank you for putting forward an interesting question. We were not interested in patients with proactive treatment since we focused on whether the activity (macular fluid) disappeared due to the anti-VEGF injection. We have revised the sentence accordingly (p. 4, lines 80–81).

***(p. 4, lines 80–81)*** (3) no anti-VEGF injections despite macular fluid – i.e., proactive treatment;

Line 83-84: Do you have proof for this A-V communication?

🡪 Thank you for pointing that out. “A-V communication” means that there was enough space through which the aqueous and vitreous humor could diffuse into each other. We thought that the presence or absence of posterior capsule represented this point. However, even with the posterior capsule acting as an aqueous-vitreous barrier, drug diffusion and a lack of A-V communication could not be completely prevented. Therefore, instead of using the term “with or without A-V communication,” we directly described the presence or absence of the posterior capsule. (i.e., with or without posterior capsule). We also changed the text to show the overall result without separating the patients into two groups and then showed the subgroup analysis results according to the presence or absence of the posterior capsule. (p. 5, lines 108–109)

***(p. 5, lines 108–109)*** A subgroup analysis was performed by comparing patients with versus those without posterior capsule.

Line 85: “Before treatment..” – which treatment vitrectomy or anti-VEGF treatment?

🡪 This refers to the first anti-VEGF treatment after vitrectomy. We have reflected on your comment by revising this sentence for clarity (p. 4, line 83).

***(p. 4, line 83)*** Before the first anti-VEGF injection after vitrectomy, …

Line 90: Which VA did you use, the best measured VA or an average or?

🡪 We did not use average visual acuity or the maximum visual acuity measured during the study period. In most cases, we used the best corrected visual acuity immediately before treatment and the visual acuity at 1 month after treatment. We have reflected your comment by adding this point (p. 5, lines 104–106; p. 10, lines 185–185).

***(p. 5, lines 104–106)*** VA was collected with the CFT and converted to the logarithm of the minimum angle of resolution (logMAR) before the analysis.

***(p. 10, lines 185–186)*** Uncorrected VA was used in only 2 cases since the best corrected VA was not measured.

Line 95: When was the first anti-VEGF injection given?

🡪 The first anti-VEGF injection was administered to a treatment-naïve patient within 2 weeks after the diagnosis of nAMD. The first anti-VEGF injection was administered to a non-treatment-naïve patient within 2 weeks after macular fluid was observed. We have added a new sentence to clarify this point (p. 5, lines 94–97).

***(p. 5, lines 94–97)*** The first anti-VEGF injection was administered to a treatment-naïve patient within 2 weeks after the diagnosis of nAMD. The first anti-VEGF injection was administered to a non-treatment-naïve patient within 2 weeks after macular fluid was observed.

Did you always use monthly injections, or PRN injections or?

🡪 As described in lines 98–101, if the macular fluid was not dried up at 1 month after the injection, an additional injection was given and the examination repeated 1 month later. Therefore, it could be said that the PRN (reactive) strategy was conducted.

***(p. 5, lines 98–101)*** Based on OCT findings, patients who showed an insufficient response were given monthly multiple injections until a sufficient decrease in intraretinal or subretinal fluid – i.e. fluid-free macula – was achieved.

Line 108: “… three groups…” Weren`t there only 2 groups, group A and B?

🡪 Analysis of variance (ANOVA) was used to compare the three subgroups. The P values in Table 3 (three groups according to choroidal neovascularization type) and Table 4 (three groups according to anti-VEGF agent type) were calculated using ANOVA.

*Results:*

Table 1: Please comment in your discussion on the amount of patients having diabetes mellitus and cancer. Could this influence your results?

🡪 The presence of diabetes mellitus or cancer did not affect anti-VEGF use.

Did you switch between different types of anti-VEGF?

🡪 Only 2 patients received bevacizumab and ranibizumab due to drug switching. The other 42 patients received only one type of anti-VEGF agent. One patient received four monthly injections (1 of ranibizumab, 3 of bevacizumab), while the other received five monthly injections (3 of ranibizumab, 2 of bevacizumab). However, this switching was not due to the ineffectiveness of ranibizumab; rather, ranibizumab was not covered by insurance since their visual acuities after treatment did not fulfill the reimbursement criteria. Therefore, the drug was switched to bevacizumab, which could be used regardless of insurance coverage.

Figure 1: N=141? On line 69 under “Materials and Methods” you have reviewed 207 medical records?

🡪 Thank you for identifying this problem. The total of N = 141 is correct. We agree with you and have reflected on your comment by correcting this number in the Materials and Methods section (p. 3, line 69–70).

***(p. 3, line 69–70)*** We retrospectively reviewed the electronic medical records of 141 vitrectomized eyes of 141 patients with nAMD treated between March 2009 and December 2018.

In the same figure you state that “Group A: With A-V communication” and Group B: Without A-V communication? Isn`t it the opposite?

🡪 Thank you for pointing out the error. The expression about the A-V communication of Groups A and B was reversed. As mentioned in the answer to the question for line 27, instead of using the term “with or without A-V communication”, we directly described the presence or absence of the posterior capsule (i.e., with or without posterior capsule). We also changed the text to show the overall result without separating the patients into two groups first and then show the subgroup analysis results according to posterior capsule status. We incorporated this in Figure 1.

Why did you exclude N=11 eyes receiving anti-VEGF for maintenance?

🡪 As mentioned above, we excluded 11 eyes that received proactive anti-VEGF treatment since we could not directly evaluate the treatment response after the anti-VEGF injections. Please see the answer to the question for line 79.

*Discussion*:

It could improve the work having a control group. Could you comment on that?

🡪 Thank you for providing these insights. Please see our answer to the first question.

Line 245: What would be the normal improvement of vision in non-vitrectomized eyes having nAMD?

🡪 That is a very important perspective. We have added the results of real-world studies that enrolled non-vitrectomized eyes to reflect your comment (p. 13, lines 254–256).

***(p.13, lines 254–256)*** In a recent real-world study of anti-VEGF with ranibizumab treatment, the mean VA change was -0.062 ± 0.33 logMAR at 1 year from baseline in treatment-naïve patients with a mean of 5.0 ± 2.7 injections.

Did your vitrectomized patients receive more injection than in general?

🡪 That is a key question. Our study could only suggest that anti-VEGF was effective in vitrectomized eyes. It was difficult to directly compare this result with that in non-vitrectomized eyes due to difficulty designing a comparative group as mentioned above (first question). Instead, we added the mean number of injections in 1 year and the VA at 1 year after the first injection to compare our result with that of the other real-world study (p. 6, lines 131–132; p. 10, line 181; p. 13–14, lines 254–258).

***(p. 6, lines 131–132)*** The total mean anti-VEGF injections was 4.14 ± 2.12 at 1 year after the first injection.

***(p. 10, line 181)*** Mean LogMAR VA was 0.90 ± 0.66 at 1 year after the first injection.

***(p. 13–14, lines 254–258)*** In a recent real-world study of anti-VEGF with ranibizumab treatment, the mean VA change was -0.062 ± 0.33 logMAR at 1 year from baseline in treatment-naïve patients with a mean of 5.0 ± 2.7 injections.[31] In our study, the mean VA change was -0.0090 ± 0.36 logMAR at 1 year from baseline with a mean 4.14 ± 2.12 injections. Most eyes maintained their VA, while only one-third showed slight post-treatment VA improvements.

We also modified Figures 3 and 5 to bar graphs to help readers better understand our results.

Thank you again for the opportunity to strengthen our manuscript with your valuable comments and queries. We have worked hard to incorporate your feedback and hope that these revisions persuade you to accept our submission.
