# Supplementary material for: Efficacy of anti-vascular endothelial growth factor agents for treating neovascular age-related macular degeneration in vitrectomized eyes
Source: PLoS One. 2021 Jun 10;16(6):e0252006. doi: 10.1371/journal.pone.0252006 (PMC8191940; doi:10.1371/journal.pone.0252006)
Supplement: Attachment — Submitted filename: Response_to_Reviewers_20210215_PlosOne.docx [file pone.0252006.s003.docx]

February 15, 2021

Manuel Alberto de Almeida e Sousa Falcão, M.D., Ph.D.

Academic Editor

*PLOS ONE*

Dear Dr. Manuel Alberto de Almeida e Sousa Falcão,

Thank you for inviting us to submit a revised draft of our manuscript entitled, "Efficacy of anti-vascular endothelial growth factor agents for treating neovascular age-related macular degeneration in vitrectomized eyes" to *PLOS ONE*. We also appreciate the time and effort you and the reviewer dedicated to providing insightful feedback about strengthening. Thus, it is with great pleasure that we resubmit our article for further consideration. We have incorporated changes that reflect the detailed suggestions you have graciously provided. We hope that our edits and the responses we provide below satisfactorily address all the issues and concerns you and the reviewer noted.

To facilitate review of our revisions, here we provide point-by-point response to the questions and comments delivered in your letter dated Jan 25, 2021.

**Reviewer #1**

Thank You so much for answering all my comments so thoroughly. It has strengthened the manuscript a lot and is now more readable. I have some small comments for this new manuscript.

🡪 Thank you for your comment. Changes made in the revised manuscript have been highlighted in yellow.

In line 22: the word using should be exchanged to with.

🡪 We have changed “using” to “with” accordingly.

***(p. 2, lines 22-23)*** The medical records were reviewed of nAMD patients treated with anti-VEGF agents who previously underwent pars plana vitrectomy (PPV).

In line 70: there is missing an a in the word treated.

🡪 We have changed “treted” to “treated” accordingly.

***(p. 4, lines 69-70)*** We retrospectively reviewed the electronic medical records of 141 vitrectomized eyes of 141 patients with nAMD treated between March 2009 to December 2018

In line 100: 2 words are written together (untila).

🡪 We have added a space between “until” and “a” accordingly.

***(p. 5, lines 101-104)*** Based on OCT findings, patients who showed an insufficient response were administered repeated injections monthly until a sufficient decrease in intraretinal or subretinal fluid, i.e., fluid-free macula, was achieved.

Using the description of with or without posterior capsule is much better than AV communication. Instead of with and without posterior capsule you could consider presence of or absence of. Are your sure that the posterior capsule always is intact when it is present?

🡪 Posterior capsulotomies were performed in some cases in the group with posterior capsule. However, even in these cases, the peripheral posterior capsules were present and firmly attached to the intraocular lenses because the openings were created in the center of the capsules. The tight attachment between the peripheral posterior capsule and the intraocular lens could act as a good barrier between the anterior and posterior chambers. This could prevent anti-VEGF agents from moving to the anterior chamber. In this respect, the posterior capsules were intact in the group with posterior capsule. We have revised the text by adding the answer to your question in the Discussion section.

***(p. 13-14, lines 244-246)*** The tight attachment between the peripheral posterior capsule and the intraocular lens could act as a good barrier in the group with posterior capsule despite posterior capsulotomy.

The very thorough explanation you are given me as a reviewer about switching between types of anti-VEGF you should consider adding in your manus or in table 1.

🡪 Thank you for raising this important question. We have revised Table 1 by adding a footnote about switching anti-VEGF agents.

***(p. 6-8, lines 140-145)* Table 1.** **Demographic and baseline characteristics of the 44 study participants**

| First anti-VEGF agent | Bevacizumab | … |
| --- | --- | --- |
|  | Ranibizumab^a^ | … |
|  | Aflibercept | … |

VEGF = vascular endothelial growth factor; RRD = Rhegmatogenous retinal detachment; VH = vitreous hemorrhage

^a^One patient received 3 bevacizumab injections after 1 ranibizumab injection and the other patient received 2 bevacizumab injections after 3 ranibizumab injections due to insurance coverage.

**Reviewer #2**

This is an interesting paper but some points need to be clarified.

🡪 Thank you for your thorough review and suggestions. Changes made in the revised manuscript have been highlighted in yellow.

It is not clear how many patients were already being treated for wet AMD at the time of the vitrectomy. I think these are the non-naïve patients, but this needs to be confirmed. But this is a very important aspect.

🡪 You have raised an important question: Twenty-seven eyes (61.4%) were non-treatment-naïve, as mentioned in lines 133–134.

***(p. 6, lines 133-134)*** Twenty-seven eyes (61.4%) were non-treatment-naïve and 2 eyes (4.5%) received photodynamic therapy before enrolling in our study.

Non-naïve patients are patients that are already being treated for their wet AMD and therefore one would not expect them to improve vision. In wet AMD, patients usually improve vision after the first 2-3 injections so we would not expect them to improve. Unless they were the active after the vitrectomy and this is a completely different situation? Were they being undertreated?

🡪 Neovascular age-related macular degeneration (nAMD) was treated the same manner in vitrectomized and non-vitrectomized eyes. Anti-vascular endothelial growth (VEGF) injection was initiated when the lesion was active. As mentioned in lines 101–104, the treatment response was monitored by optical coherence tomography (OCT) at 1 month after each injection, and patients who showed an insufficient response were given monthly multiple injections until a sufficient decrease in intraretinal or subretinal fluid, i.e., fluid-free macula, was achieved. Therefore, nAMD in vitrectomized eyes is rarely undertreated. A treat-and-extent (T&E) or pro re nata (PRN) regimen was used after fluid-free macula was achieved. Three loading doses monthly for the first 3 months were administered to treatment-naïve patients. However, there might be discrepancies between our study and randomized control trials based on intensive treatment such as monthly fixed dosing of anti-VEGF as our study used real-world data. We have clarified this point in the revised manuscript.

***(p. 5, lines 97-100)*** The treatment protocol was identical for vitrectomized and non-vitrectomized patients; 3 monthly loading injections were administered initially to treatment-naïve patients. Thereafter, patients received anti-VEGF injections pro re nata or according to a treat-and-extend regimen at the physician’s discretion.

The authors should have analyzed the non-naïve and naive patients as different subgroups….

🡪 We agree with your assessment and have added results of the subgroup analysis by treatment-naïve status.

***(p. 5, lines 110-112)*** Subgroup analyses were performed by comparing patients with and without posterior capsule as well as treatment-naïve patients and non-treatment-naïve patients.

***(p. 10-11, lines 204-210)*** In the subgroup analysis of the posterior capsule, the mean CFT of eyes with posterior capsule (n = 38) decreased significantly after the first injection (P < 0.001) and monthly multiple injections (P < 0.001). There was no significant change in baseline VA after the first injection (P = 0.714) or monthly multiple injections (P = 0.877). The mean CFT of eyes without posterior capsule (n = 6) did not significantly improve at 1 month after the first injection (P = 0.917) or monthly multiple injections (P= 0.249). There was no significant difference in VA after the first injection (P = 0.141) or monthly multiple injections (P = 0.785) (Table 5).

***(p. 11-12, lines 212-216)*** **Table 5. Comparison of central foveal thickness (CFT) and logarithm of the minimum angle of resolution (LogMAR) visual acuity (VA) between patients with and without posterior capsule as well as treatment-naïve patients and non-treatment-naïve patients**

|  |  | Baseline | 1 month after  first injection | 1 month after  monthly multiple injection |
| --- | --- | --- | --- | --- |
| With posterior capsule  (N = 38) | CFT  (mean ± SD) | 481.82 ± 147.82 μm | 413.45 ± 135.99 μm  (85.8% of baseline)  (P < 0.001^a^) | 384.68 ± 140.31 μm  (79.8% of baseline)  (P<0.001^a^) |
|  | LogMAR VA  (mean ± SD) | 0.92 ± 0.61 | 0.90 ± 0.68  (*P* = 0.714^a^) | 0.93 ± 0.69  (*P* = 0.877^a^) |
| Without posterior capsule  (N = 6) | CFT  (mean ± SD) | 457.50 ± 222.45 μm | 419.33 ± 200.80 μm  (91.6% of baseline)  (*P* = 0.917^b^) | 399.83 ± 161.67 μm  (87.4% of baseline)  (*P* = 0.249^b^) |
|  | LogMAR VA  (mean ± SD) | 0.62 ± 0.55 | 0.83 ± 0.56  (*P* = 0.141^b^) | 0.61 ± 0.59  (*P* = 0.785^b^) |
| Treatment-naïve  (N = 17) | CFT  (mean ± SD) | 537.88 ± 161.33 μm | 452.41 ± 161.67 μm  (84.1% of baseline)  (P < 0.001^a^) | 420.18 ± 143.54 μm  (78.1% of baseline)  (P<0.001^a^) |
|  | LogMAR VA  (mean ± SD) | 0.77 ± 0.64 | 0.63 ± 0.56  (*P* = 0.220^a^) | 0.66 ± 0.56  (*P* = 0.074^a^) |
| Non-treatment-naïve  (N = 27) | CFT  (mean ± SD) | 441.11 ± 144.75 μm | 390.22 ± 128.22 μm  (88.5% of baseline)  (*P* = 0.002^a^) | 365.70 ± 138.63 μm  (82.9% of baseline)  (*P* < 0.001^a^) |
|  | LogMAR VA  (mean ± SD) | 0.99 ± 0.60 | 1.04 ± 0.68  (*P* = 0.383^a^) | 1.04 ± 0.70  (*P* = 0.423^a^) |

^a^Paired t-test

^b^Wilcoxon signed-rank test

***(p. 15, lines 278-281)*** However, in our study, there was no significant change in VA after treatment in treatment-naïve eyes (N = 17). This result may be attributed to 5 eyes (29.4%) with breakthrough vitreous hemorrhage from subretinal hemorrhage, which is related with a poor visual prognosis.

Also, we do not know what were the reasons for vitrectomy in both of these groups and this also has implications on vision... were they retinal detachments? macular holes? epirretinal membranes? vitreous haemorrhage from previous injection?

🡪 We agree with your comment. We have revised Table 1 by adding treatment-naïve and non-treatment-naïve groups accordingly.

***(p. 6-8, lines 140-145)* Table 1.** **Demographic and baseline characteristics of the 44 study participants.**

|  |  | Total  (N=44) | With posterior capsule  (N=38) | Without posterior capsule  (N=6) | Treatment-naïve  (N=17) | Non-treatment-naïve  (N=27) |
| --- | --- | --- | --- | --- | --- | --- |
| Age: vitrectomy (years) |  | 71.16 ± 7.41 | 71.87 ± 7.34 | 66.67 ± 6.77 | 69.82 ± 6.85 | 72.00 ± 7.75 |
| Age: first anti-VEGF injection (years) |  | 72.39 ± 6.75 | 72.68 ± 7.08 | 70.50 ± 4.04 | 72.47 ± 4.95 | 72.33 ± 7.76 |
| Sex (male:female) |  | 24:20 | 21:17 | 3:3 | 8:9 | 16:11 |
| Diabetes mellitus |  | 29.5% (13) | 28.9% (11) | 33.3% (2) | 35.3% (6) | 25.9% (7) |
| Hypertension |  | 43.2% (19) | 44.7% (17) | 33.3% (2) | 35.3% (6) | 48.1% (13) |
| Other systemic disease | Hyperlipidemia | 9.1% (4) | 10.5% (4) | 0.0 % (0) | 11.8% (2) | 7.4% (2) |
|  | Cancer | 9.1% (4) | 7.9% (3) | 16.7% (1) | 17.6% (3) | 3.7% (1) |
| Cause of vitrectomy | RRD | 2.3% (1) | 2.6% (1) | 0.0% (0) | 5.9% (1) | 0.0% (0) |
|  | Intraocular lens dislocation | 2.3% (1) | 0.0% (0) | 16.7% (1) | 5.9% (1) | 0.0% (0) |
|  | Macular epiretinal membrane | 29.5% (13) | 34.2% (13) | 0.0% (0) | 35.3% (6) | 25.9% (7) |
|  | Breakthrough VH | 34.1% (15) | 39.5% (15) | 0.0% (0) | 29.4% (5) | 37.0% (10) |
|  | Subretinal hemorrhage | 13.6% (6) | 15.8% (6) | 0.0% (0) | 11.8% (2) | 14.8% (4) |
|  | Vitreomacular traction syndrome | 2.3% (1) | 2.6% (1) | 0.0% (0) | 0.0% (0) | 3.7% (1) |
|  | Posterior capsular rupture | 11.4% (5) | 0.0% (0) | 83.3% (5) | 5.9% (1) | 14.8% (4) |
|  | Macular hole | 4.5% (2) | 5.3% (2) | 0.0 % (0) | 5.9% (1) | 3.7% (1) |
| Choroidal neovascularization type | Type 1 | 77.3% (34) | 78.9% (30) | 66.7% (4) | 82.4% (14) | 74.1% (20) |
|  | Type 2 | 13.6% (6) | 10.5% (4) | 33.3% (2) | 5.9% (1) | 18.5% (5) |
|  | Type 3 | 6.8% (3) | 7.9% (3) | 0.0% (0) | 5.9% (1) | 7.4% (2) |
|  | Unknown | 2.3% (1) | 2.6% (1) | 0.0% (0) | 5.9% (1) | 0.0% (0) |
| Lens status | Phakia | 15.9% (7) | 18.4% (7) | 0.0% (0) | 5.9% (1) | 22.2% (6) |
|  | Pseudophakia | 84.1% (37) | 81.6% (31) | 100.0% (6) | 94.1% (16) | 77.8% (21) |
| Interval between vitrectomy and first anti-VEGF injection | <3 mo | 65.9% (29) | 71.1% (27) | 33.3% (2) | 47.1% (8) | 77.8% (21) |
|  | 3-6 mo | 4,5% (2) | 5.3% (2) | 0.0% (0) | 0.0% (0) | 7.4% (2) |
|  | 6-12 mo | 2.3% (1) | 2.6% (1) | 0.0% (0) | 0.0% (0) | 3.7% (1) |
|  | >1 year | 27.3% (12) | 21.1% (8) | 66.7% (4) | 52.9% (9) | 11.1% (3) |
| Number of monthly multiple injections | 1 | 29.5% (13) | 31.6% (12) | 16.7% (1) | 11.8% (2) | 40.7% (11) |
|  | 2 | 25.0% (11) | 26.3% (10) | 16.7% (1) | 17.6% (3) | 29.6% (8) |
|  | 3 | 34.1% (15) | 28.9% (11) | 66.7% (4) | 64.7% (11) | 14.8% (4) |
|  | 4 | 9.09% (4) | 10.5% (4) | 0.0% (0) | 0.0% (0) | 14.8% (4) |
|  | 5 | 2.27% (1) | 2.63% (1) | 0.0% (0) | 5.9% (1) | 0.0% (0) |
| First anti-VEGF agent | Bevacizumab | 43.2% (19) | 47.4% (18) | 16.7% (1) | 29.4% (5) | 51.9% (14) |
|  | Ranibizumab^a^ | 29.5% (13) | 28.9% (11) | 33.3% (2) | 35.3% (6) | 25.9% (7) |
|  | Aflibercept | 27.3% (12) | 23.7% (9) | 50.0% (3) | 35.3% (6) | 22.2% (6) |

Having a subgroup analysis of just 6 patients should be avoided as it is a very small number of patients and no valid conclusions can be obtained. Maybe they should have been left out or not directly compared.

🡪 Thank you for pointing this out. We completely agree with you. We have incorporated your comments by adding a statement regarding the difficulty of direct comparison between the two groups in the Discussion section.

***(p. 16, lines 305-308)*** In addition, since the group without posterior capsule had a very small number of patients (n = 6), a direct comparison between the groups with and without posterior capsule was not possible due to low statistical power.

It is not clear what the authors mean by no posterior capsule? Were these patients aphakic? Were they anterior chamber IOLs? Scleral suture? in the bag IOLs that had previous been treated with capsulotomy? This needs to be clarified as well.

🡪 Thank you for your suggestion. We have revised the text by adding the intraocular lens status accordingly.

***(p. 13, lines 241-244)*** Eyes without posterior capsule, such as pseudophakic eyes with sulcus-fixated or scleral-fixated intraocular lenses, had a disrupted or no posterior capsule through which undisturbed fluidic movement was possible between the anterior and posterior chambers.

Again, thank you for giving us the opportunity to strengthen our manuscript with your valuable comments and queries. We have worked hard to incorporate your feedback and hope that these revisions persuade you to accept our submission.
