# Supplementary material for: Efficacy of anti-vascular endothelial growth factor agents for treating neovascular age-related macular degeneration in vitrectomized eyes
Source: PLoS One. 2021 Jun 10;16(6):e0252006. doi: 10.1371/journal.pone.0252006 (PMC8191940; doi:10.1371/journal.pone.0252006)
Supplement: Attachment — Submitted filename: Response_to_Reviewers_20210413.docx [file pone.0252006.s004.docx]

April 13, 2021

Manuel Alberto de Almeida e Sousa Falcão, M.D., Ph.D.

Academic Editor

*PLOS ONE*

Dear Dr. Manuel Alberto de Almeida e Sousa Falcão,

Thank you for inviting us to submit a revised draft of our manuscript titled, "Efficacy of anti-vascular endothelial growth factor agents for treating neovascular age-related macular degeneration in vitrectomized eyes" to *PLOS ONE*. We also appreciate the time and effort you and the reviewer dedicated to providing insightful feedback about strengthening. Thus, it is with great pleasure that we resubmit our article for further consideration. We have incorporated changes that reflect the detailed suggestions you have graciously provided. We hope that our edits and the responses we provide below satisfactorily address all the issues and concerns you and the reviewer noted.

To facilitate review of our revisions, here we provide point-by-point response to the questions and comments delivered in your letter dated March 28, 2021.

**Reviewer #1**

Indeed a thorough and good revision. All my comments have been addressed. It has strengthened the manus to discriminate between treatment-naïve and non-treatment-naïve eyes.

You can consider to add "intact" whenever using "with posterior capsule".

🡪 Thank you for your suggestion. We also considered the word “intact” at the time of revision. However, this word can be misunderstood as the absence of defects in the posterior capsule. As mentioned on page 14, line 246, some patients might have undergone posterior capsulotomy although the tight attachment between the peripheral posterior capsule and the intraocular lens guaranteed no change of drug clearance compared to the intact posterior capsules. Therefore, we have decided not to use the word “intact.” Once again, thank you very much your kind suggestion.

You can consider to rewrite the sentence on line 87-88, exchange which with where, add VA and omit the word "together": "In cases where best corrected VA was not measured, uncorrected VA was collected."

🡪 We have changed the sentence according to your and another reviewer’s suggestions.

**(p. 4, lines 89-90)** Two cases without best-corrected VAs were excluded from the visual outcome analysis.

**Reviewer #2**

Reviewer #2: Thank you for addressing all my comments. The paper has become clearer now but there are still some points that I have now understood and need further clarification.

Firstly, please remove patients with uncorrected visual acuity. I am sorry for not having seen this in the previous review. This is not acceptable. Non corrected visual acuity has a ceiling affect that cannot used in scientific reports.

🡪 Thank you for pointing this out. Owing to its retrospective nature, best-corrected visual acuities (BCVAs) could not be collected in two cases as mentioned in page 10, lines 192 and 193. We have deleted the two cases from the dataset for the visual outcome analysis and performed an analysis on only those cases with BCVA. However, for the analysis of anatomic outcomes, such as central foveal thickness on OCT, all cases were included.

**(p. 2, lines 29-32)** The mean logarithm of the minimum angle of resolution VA was 0.85 ± 0.578 at baseline, 0.86 ± 0.63 after the first injection, and 0.84 ± 0.64 after monthly multiple injections. VA improved in 39.5% at 1 month after first injection and 45.2% at 1 month after monthly multiple injections.

**(p. 4, lines 89-90)** Two cases without best-corrected VAs were excluded from the visual outcome analysis.

**(p. 11, lines 188-195)** In patients with best-corrected VA (n = 42), mean LogMAR VA was 0.85 ± 0.57 at baseline, 0.86 ± 0.63 at 1 month after the first injection, and 0.84 ± 0.64 at 1 month after monthly multiple injections. There was also no significant difference in VA between pre- and post-injection (P = 0.985 and 0.911, respectively). Mean LogMAR VA was 0.83 ± 0.61 at 1 year after the first injection. Of the 38 eyes (those of 4 eyes were missing), VA was improved in 15 eyes (39.5%), maintained in 12 eyes (31.6%), and deteriorated in 11 eyes (28.9%) at 1 month after the first injection. Of the 42 eyes, VA was improved in 19 eyes (45.2%), maintained in 14 eyes (33.3%), and deteriorated in 9 eyes (21.4%) at 1 month after monthly multiple injections.

**(p. 11, lines 197-198) Fig 5. Change in visual acuity (VA).** The bars represent mean LogMAR VA and the error bars represent standard error of mean.

**(p. 11, lines 202-203)** There was no significant change in VA (n = 36) after the first injection (P = 0.714) or monthly multiple injections (P = 0.942).

**(p. 11, lines 209-210)** However, there was no significant change in VA (n = 16) after the first injection (P = 0.181) or monthly multiple injections (P = 0.071).

**(p. 11-12, lines 212-213)** however, there was no significant change in VA (n = 26) after the first injection (P = 0.384) or monthly multiple injections (P = 0.423) (Table 5).

**(p. 11, lines 215-221) Table 5. Comparison of central foveal thickness (CFT) and logarithm of the minimum angle of resolution (LogMAR) visual acuity (VA) between patients with and without posterior capsule as well as treatment-naïve patients and non-treatment-naïve patients**

|  |  | Baseline | 1 month after  the first injection | 1 month after  monthly multiple injections |
| --- | --- | --- | --- | --- |
| With posterior capsule  (N = 38) | CFT  (mean ± SD) | 481.82 ± 147.82 μm | 413.45 ± 135.99 μm  (85.8% of baseline)  (P < 0.001^a^) | 384.68 ± 140.31 μm  (79.8% of baseline)  (P<0.001^a^) |
|  | LogMAR VA^c^  (mean ± SD) | 0.89 ± 0.57 | 0.86 ± 0.64  (*P* = 0.714^a^) | 0.88 ± 0.64  (*P* = 0.942^a^) |
| Without posterior capsule  (N = 6) | CFT  (mean ± SD) | 457.50 ± 222.45 μm | 419.33 ± 200.80 μm  (91.6% of baseline)  (*P* = 0.917^b^) | 399.83 ± 161.67 μm  (87.4% of baseline)  (*P* = 0.249^b^) |
|  | LogMAR VA  (mean ± SD) | 0.62 ± 0.55 | 0.83 ± 0.56  (*P* = 0.141^b^) | 0.61 ± 0.59  (*P* = 0.785^b^) |
| Treatment-naïve  (N = 17) | CFT  (mean ± SD) | 537.88 ± 161.33 μm | 452.41 ± 161.67 μm  (84.1% of baseline)  (P < 0.001^a^) | 420.18 ± 143.54 μm  (78.1% of baseline)  (P<0.001^a^) |
|  | LogMAR VA^d^  (mean ± SD) | 0.70 ± 0.59 | 0.63 ± 0.57  (*P* = 0.181^a^) | 0.60 ± 0.51  (*P* = 0.071^a^) |
| Non-treatment-naïve  (N = 27) | CFT  (mean ± SD) | 441.11 ± 144.75 μm | 390.22 ± 128.22 μm  (88.5% of baseline)  (*P* = 0.002^a^) | 365.70 ± 138.63 μm  (82.9% of baseline)  (*P* < 0.001^a^) |
|  | LogMAR VA^d^  (mean ± SD) | 0.94 ± 0.55 | 0.99 ± 0.64  (*P* = 0.384^a^) | 0.99 ± 0.67  (*P* = 0.423^a^) |

^a^Paired t-test

^b^Wilcoxon signed-rank test

^c^Two cases without best-corrected visual acuities were excluded.

^d^One case without best-corrected visual acuity was excluded.

Secondly, I need to understand when first intravitreal injections were performed for most of the patients and if, in the patients that were already being treated, if anti-VEGF was used at the end of the vitrectomy.

In the 17 eyes that were treatment naive, 12 had had previous vitrectomies for other reasons. It is clear for these patients that visual acuity and OCT were measured after the vitrectomy and just before the first intravitreal treatment. And, probably, these eyes had increases in visual acuity.

However, the remaining five eyes were treated due to a breakthrough vitreous hemorrhage. This means that probably OCT was not possible to do before the vitrectomy. Also, at the time of the vitrectomy, did the surgeon perform an intravitreal anti-VEGF injection at the end of the procedure when he saw that the cause of the vitreous hemorrhage was a macular neovascularization? to avoid another breakthrough vitreous hemorrhage? If so, then the first OCT performed after the vitrectomy was not naive, but after the first injection... and therefore it really is not a baseline evaluation.

🡪 Thank you for raising the important question. In the treatment-naïve group, eyes with breakthrough vitreous hemorrhage did not receive anti-VEGF at the end of vitrectomy; therefore, OCT data at the time of the first injection could be regarded as baseline.

**(p. 5, lines 103-104)** In the treatment-naïve group, eyes with breakthrough vitreous hemorrhage did not receive anti-VEGF at the end of vitrectomy.

27 eyes that were already being treated. This means that the potential for visual recovery would be difficult because AMD patients only improve vision after the first injections. and this is an important reason for the population not having increases in visual acuity overall.

However, 16 eyes had breakthrough vitreous hemorrhages, which means that probably they were being undertreated... once again, were they not anti-VEGF treated during the vitrectomy?

🡪 In the non-treatment-naïve group, six eyes with breakthrough vitreous hemorrhage received intravitreal anti-VEGF injections at the end of vitrectomy. However, the cohort entry date of these patients was not the day of vitrectomy but the one after. Since vitreous hemorrhage made it difficult to define the pre-treatment status, such as baseline CFT, they were enrolled at the time of receiving anti-VEGF injections after vitreous hemorrhage had been resolved. We have added this point in the Methods section.

**(p. 4, 82-84 lines)** Since eyes with breakthrough vitreous hemorrhage (VH) could not be evaluated, non-treatment-naïve eyes with breakthrough VH were enrolled at the time of the first anti-VEGF injection after resolution of VH.

Please correct the abstract where it says " CFT decreased by 91.6% and 87.4% at 1 month after the first injection and after monthly multiple injections, respectively, without statistical significance."

🡪 Thank you for pointing that out. We have revised the text accordingly.

**(p. 2, lines 32-36)** In the subgroup analysis, CFT of eyes with the posterior capsule decreased significantly by 85.8% and 79.8% at 1 month after the first injection and after monthly multiple injections, respectively. CFT of eyes without the posterior capsule decreased by 91.6% and 87.4% at 1 month after the first injection and after monthly multiple injections, respectively, without statistical significance.

Please state your criteria for improving vision, stable vision or worse vision.

🡪 Improvement and worsening were defined as a change ≥0.1 LogMAR from baseline. We have added this criterion in the Methods section.

**(p. 5, lines 113-114)** Improvement and worsening of VA were defined as a change ≥0.1 LogMAR from baseline.
